# ICAM‐1‐targeted nanocarriers attenuate endothelial release of soluble ICAM‐1, an inflammatory regulator

**DOI:** 10.1002/btm2.10050

**Published:** 2017-01-17

**Authors:** Rachel L. Manthe, Silvia Muro

**Affiliations:** ^1^ Fischell Dept. of Bioengineering University of Maryland College Park MD 20742; ^2^ Institute for Bioscience and Biotechnology Research University of Maryland College Park MD 20742

**Keywords:** endothelium, ICAM‐1, inflammation, nanocarriers, soluble ICAM‐1, targeted drug delivery

## Abstract

Targeting of drug nanocarriers (NCs) to intercellular adhesion molecule‐1 (ICAM‐1), an endothelial‐surface protein overexpressed in many pathologies, has shown promise for therapeutic delivery into and across this lining. However, due to the role of ICAM‐1 in inflammation, the effects of targeting this receptor need investigation. Since ICAM‐1 binding by natural ligands (leukocyte integrins) results in release of the “soluble ICAM‐1” ectodomain (sICAM‐1), an inflammatory regulator, we investigated the influence of targeting ICAM‐1 with NCs on this process. For this, sICAM‐1 was measured by ELISA from cell‐medium supernatants, after incubation of endothelial cell (EC) monolayers in the absence versus presence of anti‐ICAM NCs. In the absence of NCs, ECs released sICAM‐1 when treated with a pro‐inflammatory cytokine. This was reduced by inhibiting matrix metalloproteinases MMP‐9 or MMP‐2, yet inhibiting both did not render additive effects. Release of sICAM‐1 mainly occurred at the basolateral versus apical side, and both MMP‐9 and MMP‐2 influenced apical release, while basolateral release depended on MMP‐9. Interestingly, anti‐ICAM NCs reduced sICAM‐1 to a greater extent than MMP inhibition, both at the apical and basolateral sides. This effect was enhanced with time, although NCs had been removed after binding to cells, ruling out a “trapping” effect of NCs. Instead, inhibiting anti‐ICAM NC endocytosis counteracted their inhibition on sICAM‐1 release. Hence, anti‐ICAM NCs inhibited sICAM‐1 release by mobilizing ICAM‐1 from the cell‐surface into intracellular vesicles. Since elevated levels of sICAM‐1 associate with numerous diseases, this effect represents a secondary benefit of using ICAM‐1‐targeted NCs for drug delivery.

## 1 Introduction

Being at the interface between the circulation and subjacent tissues, vascular endothelial cells (ECs) contribute to important physiological functions, including transport of metabolites and signaling molecules that communicate between these two compartments, regulation of vascular tone, hemostatic function, inflammatory response, and so on.^1^ As such, ECs are important targets for therapeutic intervention. An illustration of this relevance is the fact that numerous drug delivery strategies are being explored to target pharmaceuticals to the endothelium, either for endothelial‐surface release into the circulation, uptake into ECs to treat endothelial maladies, or transport across this lining to access subjacent tissues.^2^, ^3^

Among other valuable cell‐surface markers, endothelial cell adhesion molecules (CAMs) are being actively investigated as they have shown promise in the context of endothelial targeting for drug delivery applications.^4^ By contributing to the adhesion of leukocytes to the endothelium, their extravasation into sites of disease and other associated signaling cascades, endothelial CAMs play an important role in the inflammatory processes that underlie most maladies.^4^, ^5^ An example is that of intercellular adhesion molecule‐1 (ICAM‐1), an immunoglobulin‐ (Ig)‐like transmembrane glycoprotein overexpressed on the endothelial lumen during many pathological states.^5^, ^6^, ^7^ Given these properties, targeting endothelial ICAM‐1 has been studied for both diagnostic and therapeutic applications.^8^, ^9^, ^10^, ^11^, ^12^, ^13^, ^14^

Interestingly, engagement of ICAM‐1 by nanocarriers (NCs) coated with anti‐ICAM antibodies or peptides, such as the case of liposomes, nanoparticles, dendrimers, micelles, protein conjugates, and so on, induces a series of signal cascades and actin reorganization in ECs, leading to vesicular transport both into and across these cells.^15^, ^16^, ^17^, ^18^ This is mediated via the cell adhesion molecule (CAM) pathway, a clathrin‐ and caveolae‐independent route.^17^, ^18^ The CAM pathway is effective across a broad spectrum of carrier sizes (from the nano‐ to the micro‐scale), in contrast to clathrin‐ or caveolae‐mediated pathways that associate with 100–150 nm and 50–70 nm vesicles, respectively.^18^, ^19^ This correlates well with the fact that ICAM‐1 contributes to mobilization of leukocytes,^20^ a much larger “ligand” than the natural molecular counterparts transported via clathrin or caveolar means. The signal cascades that associate with endothelial uptake of anti‐ICAM NCs involve protein kinase C, Src kinases, Rho, Ca^2+^, sphingomyelinase‐mediated generation of ceramide, and so on, which are reminiscent of those elicited on leukocyte binding to ECs during inflammation.^4^, ^17^, ^18^ Similarly, the actin reorganization observed in both events are analogous and consist of mobilization of cortical into actin stress fibers.^4^, ^17^, ^18^ Ultimately, endothelial targeting and vesicular transport via ICAM‐1 has shown promise for diagnostic imaging and drug delivery applications in the context of cancer, pulmonary, cardiovascular, genetic, and other diseases.^8^, ^9^, ^10^, ^11^, ^12^, ^13^, ^14^

However, due to the role of ICAM‐1‐mediated leukocyte adhesion and signaling in inflammation,^5^, ^6^, ^7^ the secondary effects of engaging this receptor and/or blocking it from its natural counterparts are not known. In fact, this aspect has not been carefully examined yet for most of the targeted drug delivery systems described in the literature. Given that cell‐surface receptors and markers are typically involved in complex functions and/or signaling processes important to maintaining homeostasis, it is logical to assume that engagement by targeted drug carriers could lead to either beneficial or detrimental side effects, depending on their specific patho‐physiological role. In the case of ICAM‐1, it has been postulated that engagement by antibodies or drug carriers could lower the availability of this marker for engagement by its natural “ligands” (β_2_ integrins, LFA‐1 and Mac‐1, present on the leukocyte surface),^5^ therefore, attenuating inflammation, which may render secondary benefits.^21^ Conversely, it is known that ICAM‐1 binding by leukocytes initiates pro‐inflammatory cascades, including release of cytokines, generation of reactive oxygen species, expression of other CAMs, alteration of endothelial permeability, and so on.^6^, ^7^; hence, ICAM‐1 engagement by NCs could result in similar side effects.

In this context, an interesting biological process associated with ICAM‐1 overexpression during inflammation and its engagement by leukocytes is that of endothelial release of the ectodomain of ICAM‐1, which can then circulate as the “soluble” form of this molecule (sICAM‐1).^6^, ^22^ Release of sICAM‐1 is believed to occur via cleavage of the ICAM‐1 ectodomain by proteases, such as matrix metalloproteinase‐9 (MMP‐9).^23^ Additional proteases (e.g., TNFα‐converting enzyme [TACE] and elastase) may also be directly involved in ICAM‐1 cleavage,^24^, ^25^, ^26^ or others may indirectly play a role in this process, such as in the case of matrix metalloproteinase‐2 (MMP‐2), which is involved in cleavage of pro‐MMP‐9 into the active form of this protease.^27^, ^28^

The literature describes sICAM‐1 as an inflammatory marker and regulator, which can promote the inflammatory response.^6^, ^22^ Serum sICAM‐1 appears to be low in healthy individuals, but its levels increase in many pathologies, associating with the disease progression and severity, as in the case of cancer, cardiovascular disease, immune syndromes and, generally, maladies involving chronic inflammation.^6^, ^22^ This association is not merely circumstantial, but sICAM‐1 appears functionally involved in these diseases.^22^ For instance, sICAM‐1 aids in tumor progression by promoting angiogenesis and shielding tumors from cytotoxic lymphocytes, and it promotes a pro‐inflammatory phenotype by inducing the production of factors such as MIP‐1α, IL‐6, or TNFα.^22^, ^29^, ^30^, ^31^, ^32^

Therefore, in this work we began studying aspects relative to the potential secondary effects of endothelial ICAM‐1 targeting, focusing on the influence of anti‐ICAM NCs on endothelial release of sICAM‐1. The results we obtained will help rationally inform the future design of drug carriers and selection of potential applications using this strategy.

## 2 Materials and methods

### 2.1 Antibodies and reagents

Monoclonal mouse anti‐human ICAM‐1 (clone R6.5) was from ATCC (Manassas, VA) and phycoerythrin‐labeled monoclonal mouse anti‐human ICAM‐1 (clone LB‐2) was from Santa Cruz Biotechnology (Dallas, TX). Alexa Fluor 350‐labeled goat anti‐mouse IgG was from Invitrogen (Carlsbad, CA). Nonspecific mouse IgG was from Jackson ImmunoResearch (West Grove, PA). Green Fluoresbrite® polystyrene particles (100 nm in diameter) were from Polysciences (Warrington, PA). Porous transwell inserts (1.0 µm‐pore size) were from Thermo Fisher Scientific (Waltham, MA). Human sICAM‐1 ELISA kits were from Invitrogen (Carlsbad, CA). ^125^Iodine (^125^I) and Iodogen pre‐coated tubes were from PerkinElmer (Waltham, MA) and Thermo Fisher Scientific (Waltham, MA), respectively. MMP‐9 Inhibitor I and MMP‐2 Inhibitor I were from EMD Millipore (Billerica, MA). Unless specified, all other reagents were from Sigma‐Aldrich (St. Louis, MO).

### 2.2 Preparation and characterization of anti‐ICAM NCs

Model polymer NCs were prepared by coating via surface adsorption with unlabeled or ^125^I‐labeled anti‐ICAM or non‐specific IgG onto 100 nm, green Fluoresbrite® polystyrene particles (anti‐ICAM NCs vs. IgG NCs), as described.^11^ Briefly, 10^13^ NCs/ml and 5 µM antibody were incubated for 1 hr, followed by washing and centrifugation to remove unbound antibody. Final resuspension was at 7 × 10^11^ NCs/ml in phosphate buffered saline containing 1% bovine serum albumin, followed by sonication. The diameter of coated NCs was measured using particle tracking (Nanosight LM10, Malvern Instruments; Westborough, MA), while the polydispersity index (PDI) and ζ‐potential were obtained by dynamic light scattering and electrophoretic mobility, respectively (Zetasizer Nano‐S90; Malvern Instruments; Westborough, MA). The resulting antibody coat was assessed by measuring ^125^I content of a known number of particles in a gamma counter (2470 Wizard^2^, Perkin Elmer; Waltham, MA), as described.^11^

### 2.3 Cell cultures

Human umbilical vein endothelial cells (HUVECs; Lonza Walkersville, Inc.; Walkersville, MD) were cultured in M199 (Invitrogen; Carlsbad, CA) supplemented with 15% fetal bovine serum, 2 mM l‐glutamine, 15 µg/ml endothelial cell growth supplement, 100 µg/ml heparin, 100 U/ml penicillin, and 100 µg/ml streptomycin. Cells were grown at 37°C, 5% CO_2_, and 95% relative humidity on either 1% gelatin‐coated coverslips or uncoated 1.0 µm‐pore transwell inserts. Where indicated, cells were stimulated for 16–20 h with 10 ng/ml tumor necrosis factor α (TNFα) to induce endothelial activation.^5^, ^17^

### 2.4 Binding and uptake of anti‐ICAM NCs by activated ECs

TNFα‐activated HUVECs grown on coverslips were incubated for 30 min at 37°C with green Fluoresbrite® anti‐ICAM NCs or nonspecific IgG NCs (7 × 10^10^ NCs/ml), followed by washing off unbound carriers. The cells were fixed with 2% paraformaldehyde, stained with a Texas‐Red secondary antibody to label carriers bound on the cell‐surface (not internalized) as described,^17^ and cell nuclei were stained blue with 4′,6‐diamidino‐2‐phenylindole. Samples were visualized by fluorescence microscopy using an Olympus IX81 microscope, a 60× objective (Olympus, Inc.; Center Valley, PA), and blue, green, and red fluorescence filters (1160A‐OMF, 3540B‐OMF, 4040B‐OMF; Semrock, Inc.; Rochester, NY). Images were taken using an ORCA‐ER camera (Hamamatsu; Bridgewater, NJ) and SlideBook 4.2 software (Intelligent Imaging Innovations; Denver, CO), and analyzed using Image‐Pro 6.3 (Media Cybernetics, Inc.; Bethesda, MD). As in previous studies, the total number of NCs associated per cell (total green NC signal) and the number of NCs internalized within cells (total green signal minus green signal that colocalized with “surface” red signal) were quantified. This was achieved using an algorithm that normalizes the area of specific fluorescence (over a threshold background) to the number of pixels that correspond to the size of a single NC at the magnification used.^33^

To assess NC interaction with TNFα‐activated HUVECs in transwell models, ^125^I‐anti‐ICAM NCs or ^125^I‐IgG NCs were added to the apical chamber (7 × 10^10^ NCs/ml) and incubated for 30 min at 37°C (pulse). Nonbound carriers were then removed by washing both chambers, which also eliminates any NCs that may have leaked across the cell monolayer. Cells were then incubated for additional time in carrier‐free medium up to a total of 5 h, to allow transport of pre‐bound NCs (chase). After both time points, ^125^I‐anti‐ICAM NCs or ^125^I‐IgG NCs associated with the EC layer were quantified using a gamma counter. Free ^125^I was determined using trichloroacetic acid precipitation and subtracted from these measurements, to eliminate any contribution of free tracer.^33^ The absolute number of NCs in the cell fraction was calculated from the specific ^125^I activity of the carrier preparation, as described.^16^

### 2.5 Release of sICAM‐1 by ECs

Quiescent versus TNFα‐activated HUVECs, grown on coverslips or transwells, were incubated at 37°C in the absence versus presence of anti‐ICAM NCs or IgG NCs (7 × 10^10^ NCs/ml). For transwell experiments, NCs were added to the apical chamber for 30 min (pulse), followed by washing to remove unbound NCs, and incubation for additional time in carrier‐free medium up to a total of 1 or 5 h (chases). After each time, the cell medium was collected and centrifuged at 3,000*g* for 5 min, followed by 1 min centrifugation at 17,000*g* to remove residual NCs, cells, and debris. The supernatants were used to quantify sICAM‐1 by ELISA according to the manufacturer's instructions, followed by colorimetric detection using a SpectraMax M2e microplate reader (Molecular Devices; Sunnyvale, CA) at 450 nm. Similar experiments were performed in the presence of 3 mM amiloride, which inhibits CAM‐mediated transport,^17^, ^18^ and 25 µM MMP‐9 or MMP‐2 inhibitors (MMP‐9i; MMP2i), individually or in combination (Mixed MMPi).

### 2.6 Validation of sICAM‐1 differential shedding versus diffusion in transwell models

To verify lack of diffusion (indicative of differential release) of sICAM‐1 across the EC monolayer, 2 ng/ml exogenous sICAM‐1 was added to either the apical or basolateral chambers and incubated at 37°C for 4.5 h. The amount of sICAM‐1 in each chamber was then measured by ELISA, as described above. To calculate the amount of sICAM‐1 in each chamber as a percentage of sICAM‐1 added, sICAM‐1 that was released from activated ECs during this time (obtained from control experiments where exogenous sICAM‐1 was not added) was subtracted from the readings, and then the percentage was calculated.

### 2.7 Uptake of membrane ICAM‐1 versus sICAM‐1 by activated ECs incubated with anti‐ICAM NCs

TNFα‐activated HUVECs grown on coverslips were incubated for 30 min at 37°C with green Fluoresbrite® anti‐ICAM NCs (7 × 10^10^ NCs/ml). Afterward, the cells were washed to remove unbound NCs. The cells were fixed with 2% paraformaldehyde, stained with an Alexa Fluor 350 (blue) secondary antibody to label NCs bound on the cell‐surface (not internalized), and then permeabilized with 0.1% Triton X‐100 and stained with a phycoerythrin (pseudocolored red) anti‐ICAM‐1 (clone LB‐2) antibody to label both cell‐surface and internalized NCs. Hence, cell‐surface NCs appear white (green + blue + red), internalized membrane ICAM‐1 complexed with NCs appear yellow (green + red), and internalized NCs without internalized membrane ICAM‐1 appear green alone. Images were captured as described above. Alternatively, after NC removal by washing, cells were lysed and the amount of sICAM‐1 in these cell lysates was measured by ELISA, as described above.

### 2.8 Statistics

Experiments encompass a total sample size of *n* ≥ 4. Data were calculated as the mean ± standard error of the mean (SEM). Statistical significance was determined as *p* < 0.1 by Student's *t*‐test or by Mann‐Whitney Rank Sum test, as indicated.

## 3 Results

### 3.1 Release of sICAM‐1 by ECs and differential apical versus basolateral distribution

**Figure 1:**
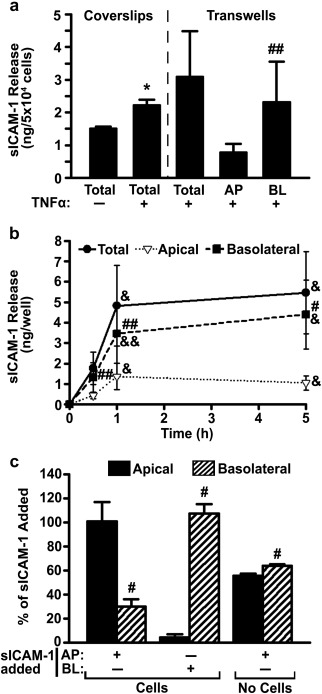
Release of sICAM‐1 by ECs. (a) HUVECs were grown on coverslips or transwell inserts in control medium versus medium containing TNFα (16 h). Cells were then washed and sICAM‐1 release into the cell medium [apical (AP), basolateral (BL), and total (AP + BL)] was examined after 30 min, using ELISA. (b) Distribution of sICAM‐1 release by TNFα‐activated HUVECs grown on transwells was similarly measured at 30 min, 1 h, or 5 h. (c) Relative distribution of exogenous sICAM‐1, 4.5 h after its addition to the AP or BL chambers of transwells in the absence of cells versus the presence of TNFα‐activated HUVECs. Data are mean ± SEM. *Comparison to non‐activated ECs; #comparison between apical and basolateral chambers at each time point; &comparison to 30 min (one symbol is *p* < 0.1 by Student's *t*‐test and two symbols is *p* < 0.1 by Mann‐Whitney Rank Sum test)

ECs increase their release of sICAM‐1 when activated during inflammation.^22^, ^23^, ^29^ Hence, we first validated our detection of this phenomenon using ECs grown on coverslips, the most common model used in prior sICAM‐1 studies in cell culture. We incubated ECs for 16 h with the pro‐inflammatory cytokine TNFα (activation pulse), then removed TNFα and continued incubations in fresh medium (release chase). As expected, TNFα enhanced sICAM‐1 release by ECs compared to nonactivated counterparts: a 1.5‐fold increase in a period of 30 min (Figure 1a). Then, we repeated this assay using ECs grown as a monolayer on transwell inserts, a model that better reflects the natural status of ECs by separating apical and basolateral compartments. Total sICAM‐1 release in this model seemed similar or slightly enhanced to the coverslip model (1.4‐fold at 30 min; Figure 1a). This setting also allowed us to independently examine sICAM‐1 release at the apical versus basolateral sides of the EC monolayer. Unexpectedly, we observed a preferential release into the basolateral chamber underneath the cells (75% of total sICAM‐1, 3‐fold over the apical fraction; Figure 1a). The release of sICAM‐1 continued increasing up to 1 h (2.7‐fold over 30 min), then it seemed to saturate (at 5 h it was 1.1‐fold over 1 h; Figure 1b). During all this time, the pattern of preferential basolateral release was maintained and, at saturation (5 h), basolateral sICAM‐1 surpassed the apical fraction by 4.2‐fold (Figure 1b).

To validate the specificity of this differential pattern, we tested the passive permeability of the EC monolayer and the transwell filter (Figure 1c). In the absence of cells, addition of exogenous sICAM‐1 to the apical chamber resulted in a similar distribution in both compartments (56% apical and 64% basolateral; Figure 1c and Supporting Information Figure S1). This indicates that sICAM‐1 can diffuse through the filter pores, as expected. Then, we added exogenous sICAM‐1 to either the apical or basolateral chambers separated by ECs, and sICAM‐1 was measured in either chamber after 4.5 h. Opposite to the pattern of sICAM‐1 release by cells, addition of exogenous sICAM‐1 to the apical side resulted in a specific increase of the sICAM‐1 level in this chamber, that is, 3.4‐fold over the basolateral side (Figure 1c and Supporting Information Figure S1). This was 6.9‐fold greater than the amount released by cells in this chamber, while detection in the basolateral chamber did not vary (1.2‐fold over that of cells alone; Supporting Information Figure S2). Conversely, exogenous sICAM‐1 addition to the basolateral chamber resulted in an even greater enhancement in this chamber (24‐fold over the apical side; Figure 1c and Supporting Information Figure S1). This addition rendered a minor increase in the level of sICAM‐1 in the apical chamber (1.5‐fold over cells alone) and a slightly greater increase in the basolateral fraction (2.3‐fold; Supporting Information Figure S2). Since cells alone release more sICAM‐1 to the basolateral side, it was expected that adding exogenous sICAM‐1 to this chamber would enhance sICAM‐1 to a lesser extent than adding it to the apical side (Supporting Information Figure S2). Yet, when comparing the absolute amount of exogenous sICAM‐1 recovered from the basolateral versus apical chambers (middle bars in Supporting Information Figure S1), it is clear that this corresponds to almost all sICAM‐1 added. These results indicate that there is minimal (if any) passive leakage of sICAM‐1 across EC monolayers and the levels of sICAM‐1 detected in this model correspond to differential basolateral and apical release by ECs.

### 3.2 MMP mechanism of sICAM‐1 release by ECs

**Figure 2:**
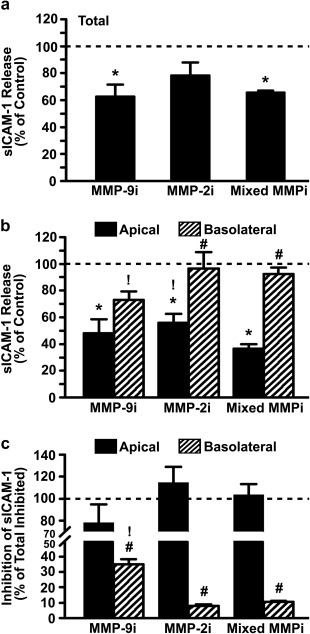
Effect of MMP inhibition on sICAM‐1 release by activated ECs. TNFα‐activated HUVECs grown on transwell inserts were incubated in control medium or medium containing inhibitors of MMP‐9 (MMP‐9i), MMP‐2 (MMP‐2i), or a mixture of both (Mixed MMPi) for 30 min. (a) Total (apical + basolateral) and (b) apical versus basolateral release of sICAM‐1, as measured by ELISA and expressed as the percentage of control without inhibitors (horizontal dashed line). (c) Inhibition of apical or basolateral release of sICAM‐1 relative to the total inhibition observed (horizontal dashed line). Data are mean ± SEM. *Comparison to control for each condition and chamber; #comparison between apical and basolateral chambers;! comparison to mixed MMPi for each chamber (*p* < 0.1 by Student's *t*‐test)

It has been reported that release of sICAM‐1 by ECs may be in part contributed by shedding of ICAM‐1 expressed at the cell‐surface, a phenomenon also observed for other endothelial CAMs.^22^, ^29^, ^30^, ^31^, ^32^ This is believed to be mediated via cleavage of the ICAM‐1 ectodomain by proteases, such as the case for matrix metalloproteinase MMP‐9.^23^ Therefore, to examine if this mechanism contributes to the observed differential distribution of sICAM‐1 across activated EC monolayers, we measured release in the presence of MMP‐9 and/or MMP‐2 inhibitors, whereby MMP‐2 was meant to serve as a control. We focused on the first 30 min after removal of the TNFα pulse, as this had shown active release rather than saturation (Figure 1b). As expected, MMP‐9 inhibition led to a 37% decrease in total sICAM‐1 by activated ECs (Figure 2a). MMP‐2 inhibition also reduced total sICAM‐1 (by 22%, not significant), and simultaneous inhibition of MMP‐9 and MMP‐2 behaved as MMP‐9 inhibition alone (34% reduction; Figure 2a).

Interestingly, compared with respective controls in each chamber, the effect caused by MMP‐9 was greater in the apical versus basolateral chamber (48% vs. 73% of respective chamber controls; Figure 2b). Comparing the absolute amount of sICAM‐1 contributed by MMP‐9 to either chamber (Figure 2c), we observed that inhibition of this proteinase more readily decreased sICAM‐1 in the apical versus basolateral chamber (78% vs. 35% contribution). MMP‐2 inhibition also decreased apical sICAM‐1 release (56% of control), although no effect was observed with regard to basolateral release (96% of control; Figure 2b). In fact, when comparing the absolute amount of sICAM‐1 contributed by MMP‐2 in these chambers, only apical activity was found (114% vs. 8% contribution; Figure 2c). Despite observing MMP‐9 and MMP‐2 effects in the apical chamber, simultaneous inhibition of both proteinases did not render enhanced or additive effects on this side (37% of control, statistically similar to MMP‐9 inhibition alone) and little inhibition arose in the basolateral chamber (92% of control; Figure 2b). Altogether, these results suggest that both MMPs are functionally involved in sICAM‐1 release to the apical space, with MMP‐9 contributing to a greater extent, while MMP‐9 alone contributes to basolateral sICAM‐1 release and this contribution is lower than that at the apical side.

### 3.3 Effect of anti‐ICAM NCs on sICAM‐1 release by ECs

In addition to pro‐inflammatory factors alone, sICAM‐1 release has been postulated to associate with other events. For instance, binding of leukocytes to endothelial CAMs is known to regulate leukocyte‐endothelial interactions during inflammation, including disengagement of both cell types after leukocyte extravasation.^23^, ^34^, ^35^, ^36^, ^37^ In this context, leukocyte binding to endothelial CAMs seems to result in both leukocyte and endothelial secretion of MMPs, as well as sICAM‐1 release.^4^ Therefore, it is plausible that binding of ICAM‐1‐targeted NCs to endothelial ICAM‐1 may result in a similar effect.

**Figure 3:**
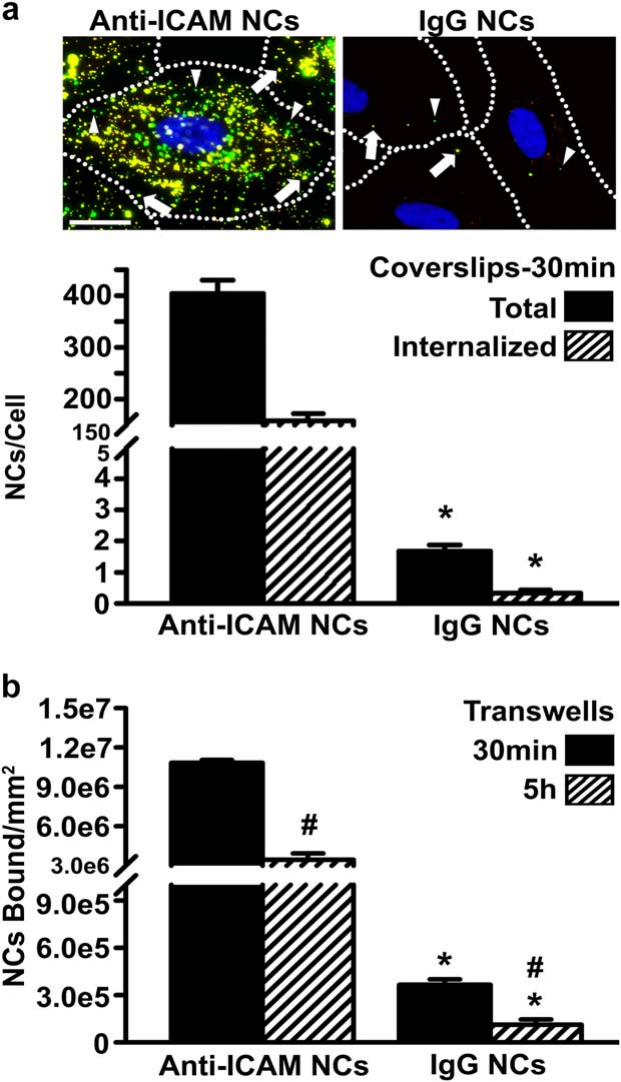
Specific interaction of anti‐ICAM NCs with activated ECs. (a) TNFα‐activated HUVECs were incubated for 30 min at 37°C with green fluorescent anti‐ICAM or IgG NCs. Non‐bound NCs were removed by washing, and surface‐bound NCs were immunostained using a Texas‐Red secondary antibody, which renders non‐internalized NCs double labeled in green + red (yellow; arrows) versus single‐labeled, green internalized NCs (arrowheads). Images and quantification of total cell‐associated (bound + internalized) and internalized NCs are shown. Scale bar = 10 µm. (b) TNFα‐activated HUVECs grown on transwell inserts were incubated for 30 min at 37°C with ^125^I‐anti‐ICAM or ^125^I‐IgG NCs. Non‐bound NCs were removed by washing and cells were incubated in fresh medium up to 5 h. NCs associated with the cell fraction were quantified using a gamma counter at both time points. Data are mean ± SEM. *Comparison between anti‐ICAM and IgG NCs; #comparison between 30 min and 5 h (*p* < 0.1 by Student's *t*‐test)

To examine this, we used model polystyrene nanoparticles coated with anti‐ICAM (anti‐ICAM NCs) versus non‐specific IgG (IgG NCs). This material is not biodegradable and, although not significant clinically, this property allows us to examine ICAM‐1 targeting and sICAM‐1 release events without confounding results that may arise from simultaneous NC degradation. As shown in our previous studies, these formulations are relatively stable (i.e., lack of: aggregation, antibody detachment, and albumin coating)^11^ and render binding, endocytosis, intracellular trafficking, and in vivo circulation and biodistribution comparable to biodegradable poly(lactic‐co‐glycolic acid) carriers,^38^, ^39^ thereby validating the use of this model. Compared to uncoated counterparts, antibody‐coated NCs had increased diameter (155 vs. 110 nm; Table 1) and ζ‐potential (−28 vs. −35 mV), while still maintaining a reasonably low PDI (0.15 vs. 0.06). In particular, anti‐ICAM NCs had a size of 157 nm, PDI of 0.18, ζ‐potential of −27 mV, and 267 antibody molecules/NC. These parameters were similar to those of nonspecific IgG NCs, which had a size of 158 nm, PDI of 0.15, ζ‐potential of −29 mV, and 193 antibody molecules/NC. As described previously,^11^, ^17^ fluorescence microscopy showed that anti‐ICAM NCs specifically bound to and were internalized by activated ECs grown on coverslips: 241‐fold and 456‐fold over nonspecific IgG NCs, respectively, after only 30 min incubation (Figure 3a). In addition, radioisotope tracing of ^125^I‐anti‐ICAM NCs showed that this formulation also bound specifically to activated ECs grown on transwell inserts, for example, 30‐fold over ^125^I‐IgG NCs after 30 min incubation (Figure 3b). A similar specificity was found after 5 h incubation (31‐fold over IgG NCs). Yet, at this time the absolute amount of NCs associated with cells decreased below that observed at 30 min (3.1‐fold decrease for anti‐ICAM NCs), which is consistent with transendothelial transport previously observed in this model.^16^

**Table 1:** NC characterization

	Size (nm)	Polydispersity index	Zeta potential (mV)	Antibodies/NC
Uncoated NCs	109.3 ± 1.8	0.06 ± 0.01	−35.5 ± 1.2	N/A
Anti‐ICAM NCs	156.8 ± 2.3	0.18 ± 0.01	−27.4 ± 0.3	267 ± 10
IgG NCs	158.0 ± 4.9	0.15 ± 0.01	−28.7 ± 0.4	193 ± 13

Data are mean ± SEM.

**Figure 4:**
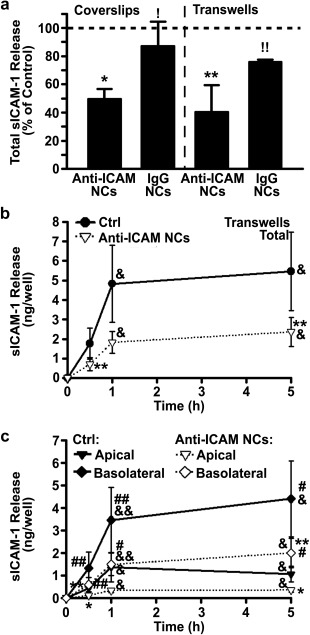
Attenuation of sICAM‐1 release by anti‐ICAM NCs. (a) Total sICAM‐1 release by TNFα‐activated HUVECs grown on coverslips or transwell inserts after incubation with anti‐ICAM NCs or non‐specific IgG NCs for 30 min. Data are presented as a percentage of cells incubated in the absence of NCs (control; horizontal dashed line). (b,c) Cumulative distribution of sICAM‐1 release by activated HUVECs grown on transwells and incubated in control medium (Ctrl) or medium containing anti‐ICAM NCs. Incubations were for 30 min (pulse), followed by NC removal and incubation in fresh medium for additional time up to 1 h or 5 h (chases). Total sICAM‐1 in (a) and (b) represents the apical + basolateral fractions. Data are mean ± SEM. *Comparison to control;! comparison between anti‐ICAM and IgG NCs; #comparison between apical and basolateral chambers at each time point; &comparison to 30 min (one symbol is *p* < 0.1 by Student's *t*‐test and two symbols is *p* < 0.1 by Mann‐Whitney Rank Sum test)

Next, we focused on the effect of anti‐ICAM NCs on sICAM‐1 release by activated ECs. As in the absence of NCs (Figure 1b), preferential release of sICAM‐1 to the basolateral versus apical side was also observed in the presence of anti‐ICAM NCs (Supporting Information Figure S3). However, surprisingly, NCs significantly inhibited this process (Figure 4): after 30 min incubation, anti‐ICAM NCs reduced by 50% total sICAM‐1 released by ECs grown in the coverslip model and by 60% in the transwell model, which was specific compared to IgG NCs (13% and 24% reduction, respectively; Figure 4a). This effect persisted with time and was observed in both the apical and basolateral compartments (Figure 4b,c and Supporting Information Figure S4).

**Figure 5:**
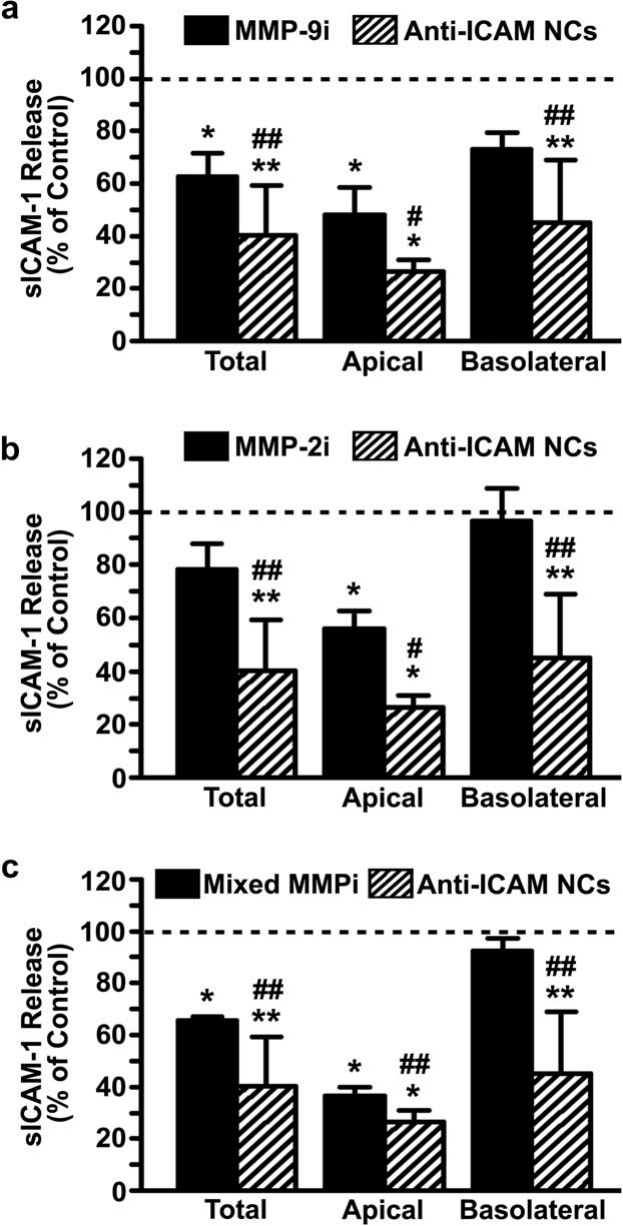
Comparative reduction of sICAM‐1 by anti‐ICAM NCs versus MMP inhibitors. Reduction in sICAM‐1 release by TNFα‐activated HUVECs grown on transwell inserts and incubated for 30 min with MMP inhibitors (MMP‐9i (a), MMP‐2i (b), or a mixture of both [Mixed MMPi] (c)) or with anti‐ICAM NCs. Data show sICAM‐1 release as a percentage of controls (absence of inhibitors and NCs; horizontal dashed line). Data are mean ± SEM. *Comparison to control; #comparison between anti‐ICAM NCs and inhibitors (one symbol is *p* < 0.1 by Student's *t*‐test and two symbols is *p* < 0.1 by Mann‐Whitney Rank Sum test)

Reduction of sICAM‐1 release by NCs was greater in the apical compartment versus the basolateral side: for example, at 30 min, respective reductions of 73% versus 55% were observed (Supporting Information Figure S4B,C). However, over time the inhibitory effect of anti‐ICAM NCs was more balanced between the two chambers (66% apical and 55% basolateral reduction by 5 h; Supporting Information Figure S4B,C). Importantly, anti‐ICAM NCs reduced release of sICAM‐1 at a greater extent than MMP inhibitors (Figure 5). At 30 min when active sICAM‐1 release was occurring, anti‐ICAM NCs reduced total sICAM‐1 by 60% compared to 37%, 22%, and 34% reductions rendered by inhibiting MMP‐9, MMP‐2, or both simultaneously. This was also the case for attenuation of sICAM‐1 at the apical side (73% reduction by NCs versus 52%, 44%, and 63% decrease for inhibitors of MMP‐9, MMP‐2, and both) and the basolateral side (55% reduction by NCs versus 27%, 4%, and 8% decrease for inhibitors of MMP‐9, MMP‐2, and both).

### 3.4 Mechanism by which anti‐ICAM NCs reduce sICAM‐1 release by ECs

A possible explanation for the inhibitory effect observed is that sICAM‐1 could be captured by anti‐ICAM NCs. Binding of sICAM‐1 to anti‐ICAM NCs would outcompete NC binding to cells and these sICAM‐1‐bound NCs would remain in the cell medium. Subsequently, they would be removed by centrifugation prior to the ELISA measurement used to detect sICAM‐1. To test this, we washed cells after the initial 30 min incubation with anti‐ICAM NCs to remove the NCs that did not bind to cells (and may contain sICAM‐1), and continued the incubation in NC‐free medium for another 30 min (1 h data shown in Figure 4b,c and Supporting Information Figure S4). Despite the absence of anti‐ICAM NCs in the milieu, the same reduction in sICAM‐1 was observed in the total, apical, or basolateral chambers (compare the first 30 min to the second 30 min (1 h) in Supporting Information Figure S4 or respective slopes in Figure 4b,c). For instance, 73% and 55% reductions were seen in the apical and basolateral sides in the presence of anti‐ICAM NCs in the milieu (first 30 min), and 75% and 57% reductions were observed if removed from the milieu (second 30 min [1 h]). Since the number of NCs interacting with cells at 30 min is the same than at 1 h (no more binding was possible since NCs had been removed from the milieu), this indicates that reduction of sICAM‐1 is not caused by anti‐ICAM NCs in the milieu, but by NCs interacting with cells.

**Figure 6:**
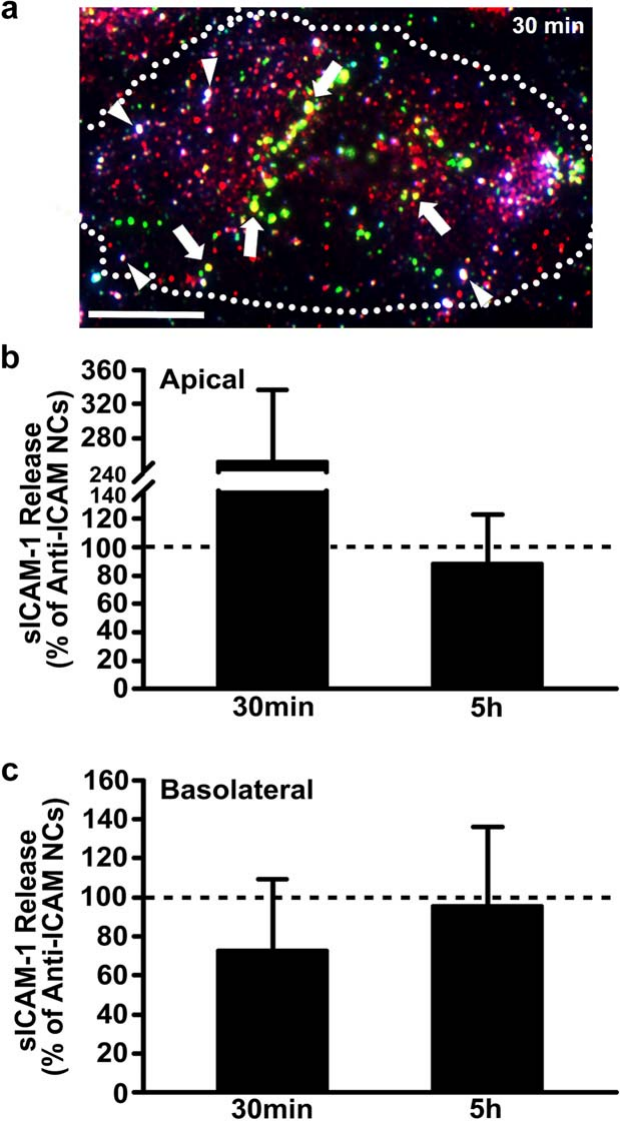
Inhibition of anti‐ICAM NC endocytosis attenuates sICAM‐1 release. (a) Image of TNFα‐activated HUVECs grown on coverslips and incubated for 30 min at 37°C with green fluorescent anti‐ICAM NCs. Nonbound NCs were removed by washing, and then the cells were fixed and immunostained (see Materials and Methods) to render surface‐bound NCs triple labeled in green + blue + red (white color; arrowheads). Instead, internalized membrane ICAM‐1 complexed with NCs appears double labeled in green + red (yellow color; arrows) and internalized NCs without membrane ICAM‐1 are labeled in green alone. Scale bar = 10 µm. (b) Apical and (c) basolateral release of sICAM‐1 by TNFα‐activated HUVECs grown on transwell inserts and incubated with anti‐ICAM NCs in the presence of amiloride, an inhibitor of CAM endocytosis. Incubations were for 30 min (pulse), followed by anti‐ICAM NC removal and incubation for additional time up to 5 h (chase). Data are expressed relative to absence of amiloride (control; horizontal dashed line). Data are mean ± SEM

Supporting this, it has been shown that when anti‐ICAM NCs bind to cell‐membrane ICAM‐1, this receptor‐NC complex is internalized, which reduces the level of ICAM‐1 displayed at the membrane^40^ (Figure 6a also validates this previous finding). Co‐uptake of membrane ICAM‐1 with NCs could then, in turn, diminish the amount of ICAM‐1 available for shedding from the plasmalemma. To test this alternative, we examined the effect of anti‐ICAM NCs on sICAM‐1 release in the presence of amiloride (Figure 6b,c), an inhibitor of the CAM pathway.^17^, ^18^ It was expected that inhibiting NC uptake would also inhibit uptake of membrane ICAM‐1 and, hence, the inhibitory effect of NCs on sICAM‐1 release would be lost. Figure 6b shows that this is the case: at 30 min when active uptake of anti‐ICAM NCs occurred (see Figure 3a), amiloride enhanced apical sICAM‐1 by 2.5‐fold compared to cells incubated with anti‐ICAM NCs in the absence of this inhibitor, while no effect of amiloride was found at 5 h (0.88‐fold) when both active sICAM‐1 release and NC endocytosis had saturated and these processes were no longer active. Instead, no increase was observed at the basolateral side at either time point (0.73‐fold and 0.95‐fold, respectively; Figure 6c). In addition, negligible amounts of sICAM‐1 were found in the cell lysates after 30 min incubation with anti‐ICAM NCs, implying that reduced sICAM‐1 release by NCs is not due to uptake of the cleaved receptor (0.003 ± 0.001 ng/well internalized sICAM‐1 versus 1.5 ± 0.3 ng/well sICAM‐1 released into the cell medium). Therefore, attenuation of sICAM‐1 release in the presence of anti‐ICAM NCs is due to removal of surface ICAM‐1 during uptake of the carrier‐receptor complex.

## 4 Discussion

Targeting of imaging agents and drug NCs to endothelial ICAM‐1 holds promise in the context of diagnostic and therapeutic interventions focused at the vascular endothelium.^8^, ^9^, ^10^, ^11^, ^12^, ^13^, ^14^ Binding of drug NCs to ICAM‐1 on the endothelial surface induces signaling conducive to NC transport into and across this lining.^16^, ^17^, ^18^ Although numerous cell culture and in vivo studies suggest that no acute toxicity associates with this strategy,^8^, ^9^, ^10^, ^11^, ^12^, ^13^, ^14^ potential side effects must be carefully examined for further translational development. This work investigated the influence of ICAM‐1 targeting on endothelial release of sICAM‐1, a marker of inflammation that associates with a variety of maladies,^22^ revealing an unexpected benefit of anti‐ICAM NCs.

During inflammation, which underlies most pathologies, ICAM‐1 becomes overexpressed on the surface of activated ECs and, concomitantly, sICAM‐1 levels increase in serum.^22^ TNFα and other pro‐inflammatory factors (IL‐1β, IL‐6, IFN‐γ, angiotensin II, etc.) induce such an outcome.^22^, ^29^, ^41^ The mechanism for sICAM‐1 release is not fully characterized, yet the soluble form of this molecule can arise from proteolytic cleavage of the ectodomain of cell‐surface ICAM‐1.^23^, ^29^, ^41^ We reproduced this by treating ECs with TNFα, using both a solid surface model and a transwell filter model. While the first model is commonly described,^24^, ^25^, ^26^, ^41^ sICAM‐1 release on polarized linings has been predominantly reported for epithelial cells, not ECs.^42^, ^43^ From our study, it appears that activated ECs separating apical and basolateral compartments released slightly greater levels of sICAM‐1 than those not polarized (Figure 1), where basolateral levels surpassed apical ones. While we could not find direct comparisons on both models in the literature, work showing independent experiments using solid‐surface and transwells seems to support our finding.^44^ Also, preferential basolateral secretion is supported by previous studies.^45^ The fact that, in vivo, sICAM‐1 has been found not only in serum but it has also been implicated in angiogenesis, migration of vascular smooth muscle cells, and other events involving the basolateral space,^6^ indicates that its differential distribution reflects a functional purpose. Hence, both apical and basolateral release events must be considered.

MMP‐9 was involved in endothelial release of sICAM‐1 on inflammatory stimulation (Figure 2), in agreement with the literature.^23^ It has been shown that ICAM‐1 provides cell‐surface docking for pro‐MMP‐9, the latent form of MMP‐9,^23^ and then the active enzyme can cleave ICAM‐1 at its membrane‐proximal domain.^23^ Yet, our results also suggest that the contribution of MMP‐9 to this event was partial and there must be other factors involved. Inhibition of MMP‐2 seemed to influence sICAM‐1 release and this could be an additional mechanism. However, simultaneous inhibition of MMP‐9 and MMP‐2 did not render an enhanced effect, ruling out this option, in accord with the fact that ICAM‐1 is not known to be an MMP‐2 substrate. Yet, MMP‐2 may indirectly play a role in this process since it can cleave pro‐MMP‐9 into MMP‐9.^27^, ^28^ This would explain why MMP‐2 inhibition resulted in a reduction of sICAM‐1 release similar to MMP‐9 inhibition, but no additive effects were found when inhibiting both (Figure 2). Hence, additional factors must contribute to sICAM‐1 release in our assays. Other studies have shown mRNA transcripts specifically encoding sICAM‐1,^46^, ^47^ but our assays involve pulse‐chase experiments to examine release within 30 min, and de novo protein synthesis is unlikely to play a major role. Other molecules that may be involved in this process include TACE and elastase, as seen previously.^23^, ^24^, ^25^, ^26^

While inhibiting MMPs was expected to decrease sICAM‐1 release, the inhibitory effect of anti‐ICAM NCs on this event (Figure 4) was unexpected, based on the fact that ICAM‐1 binding by its natural ligands, leukocytes, elicits (instead of reduces) sICAM‐1 release. This may help dynamic detachment of leukocyte‐endothelial engagement in areas where the leukocyte “samples” the endothelial surface prior to extravasation.^4^, ^23^, ^34^, ^35^, ^36^, ^37^ It may also play a role in loosening leukocyte‐endothelial attachment after extravasation and to subsequently downregulate cell‐surface ICAM‐1.^4^, ^23^, ^34^, ^35^, ^36^, ^37^ Because the signaling cascades induced in ECs by NC binding to ICAM‐1 are reminiscent of those induced by leukocytes,^4^ an increase in sICAM‐1 release was expected. However, the reduction observed can be understood based on the fact that anti‐ICAM NCs are rapidly internalized by ECs and, since they bind to ICAM‐1, their internalization removes ICAM‐1 from the plasmalemma, as we have previously shown.^40^ In fact, we have observed that most cell‐surface ICAM‐1 is internalized during this event (as verified in Figure 6a) and then, once within cells, NCs traffick to lysosomes or are transcytosed, whereas a fraction of internalized ICAM‐1 recycles back to the plasmalemma after 1 h.^15^, ^16^, ^40^ Hence, by reducing the availability of membrane ICAM‐1 on the cell‐surface, anti‐ICAM NCs reduced sICAM‐1 release. This was demonstrated by the fact that inhibiting NC uptake with amiloride counteracted the inhibitory effects on sICAM‐1 release (Figure 6b,c) and that only negligible amounts of sICAM‐1 was fund in cell lysates. In previous publications, we have shown that amiloride decreased endocytosis of anti‐ICAM NCs without affecting their binding^17^, ^18^; hence, validating this mechanism. In addition, the time and location of this inhibitory effect (predominant on the apical side at 30 min; Figure 4) pairs well with a role for anti‐ICAM NC endocytosis in lowering surface ICAM‐1 and sICAM‐1 release.

## 5 Conclusions

Given that elevated sICAM‐1 is considered a pathological marker implicated in the development of numerous pathologies (inflammation, atherosclerosis, cancers, neurological disorders, autoimmune diseases, etc.),^6^, ^22^ attenuation of its release may benefit these conditions. Interestingly, inhibition of sICAM‐1 release by anti‐ICAM NCs surpassed that of MMP inhibitors in the apical and basolateral compartments (Figure 5), which suggests translational relevance. As said, multiple factors appear to contribute to sICAM‐1 release from activated ECs and their individual inhibition very partially reduces this event. Using a cocktail of inhibitors to improve their outcome requires knowing all factors involved in each pathological situation (currently unknown), and would pose serious risk as they regulate multiple processes apart from ICAM‐1 cleavage. Instead, reducing sICAM‐1 release may be a bonus of using ICAM‐1 targeting for drug delivery applications, whereby NCs may combine the action of their therapeutic cargo with this secondary effect. Anti‐ICAM NCs reduce sICAM‐1 by decreasing cell‐surface ICAM‐1 during endocytosis, regardless of the factors involved in ICAM‐1 cleavage and without inhibiting their activity in other necessary functions. Therefore, this potentially beneficial effect deserves further attention and careful examination in animal models of disease, which we aim to investigate in the future.

## Supporting information

Additional Supporting Information may be found in the online in the supporting information tab for this article.

Supporting Information
