# Supplementary material for: ICAM‐1‐targeted nanocarriers attenuate endothelial release of soluble ICAM‐1, an inflammatory regulator
Source: Bioeng Transl Med. 2017 Jan 17;2(1):109–19. doi: 10.1002/btm2.10050 (PMC5510616; doi:10.1002/btm2.10050)
Supplement: Supplementary file 1 — Supporting Information [file BTM2-2-109-s001.pdf]

ICAM-1-Targeted Nanocarriers Attenuate Endothelial Release of Soluble ICAM-1, an Inflammatory Regulator

Rachel L. Manthe[1] and Silvia Muro[1,2,*]

[1]Fischell Department of Bioengineering and [2]Institute for Bioscience and Biotechnology Research, University of Maryland, College Park, MD 20742-4450, USA

Running head: Anti-ICAM nanocarriers reduce soluble ICAM-1

***Address correspondence to**: Silvia Muro, 5115 Plant Sciences Building, College Park, MD 20742-4450, USA. Tel: 1+301-405-4777; Fax: 1+301-314-9075; Email: muro@umd.edu

SUPPLEMENTARY INFORMATION

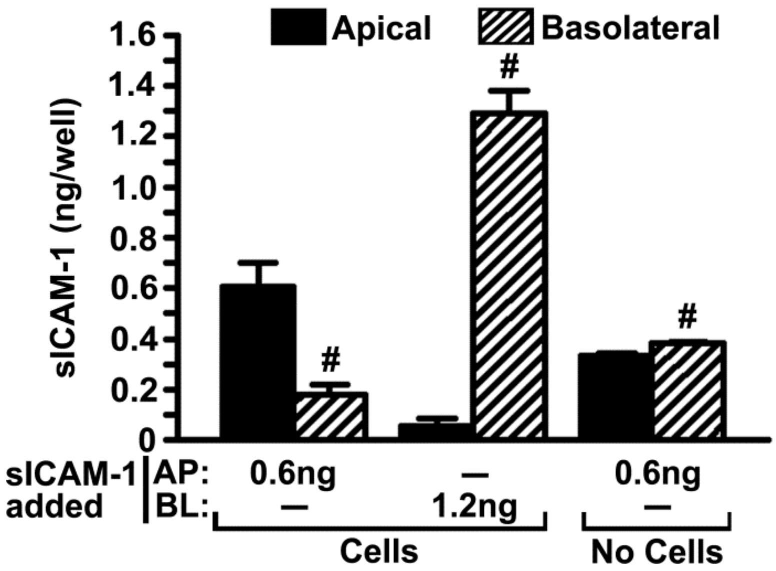

Figure S1. Exogenous sICAM-1 was added to the apical or basolateral chambers of transwell inserts where TNFα-activated HUVEC monolayers had been grown, and sICAM-1 was measured in both chambers after 4.5 h. Data show exogenous sICAM-1 detected in these chambers (total sICAM-1 in each chamber minus sICAM-1 contributed to each chamber from cellular release). Data are mean ± SEM. #Comparison between apical and basolateral chambers ($p < 0.1$ by Student's *t*-test).

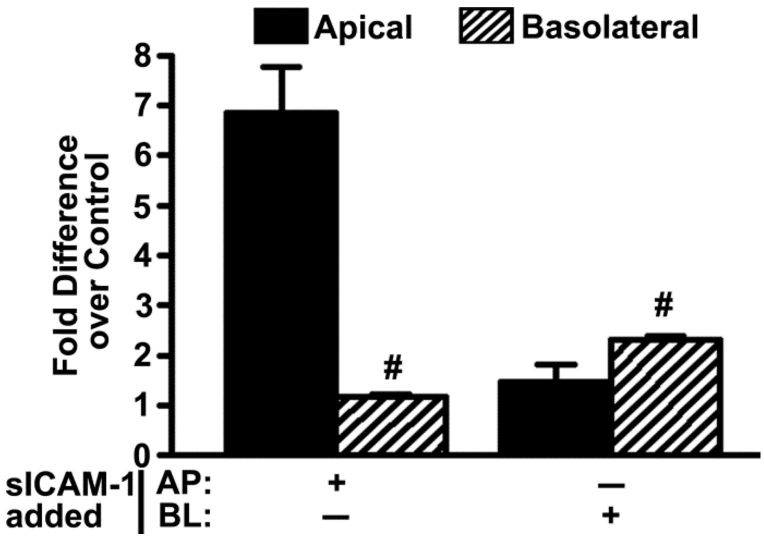

Figure S2. Exogenous sICAM-1 was added to the apical or basolateral chambers of transwell inserts where TNFα-activated HUVECs monolayers had been grown, and sICAM-1 was measured in both chambers after 4.5 h. Data show the fold difference in sICAM-1 detection over basal detection (no addition of exogenous sICAM-1) for each chamber. Data are mean ± SEM. #Comparison between apical and basolateral chambers ($p < 0.1$ by Student's *t*-test).

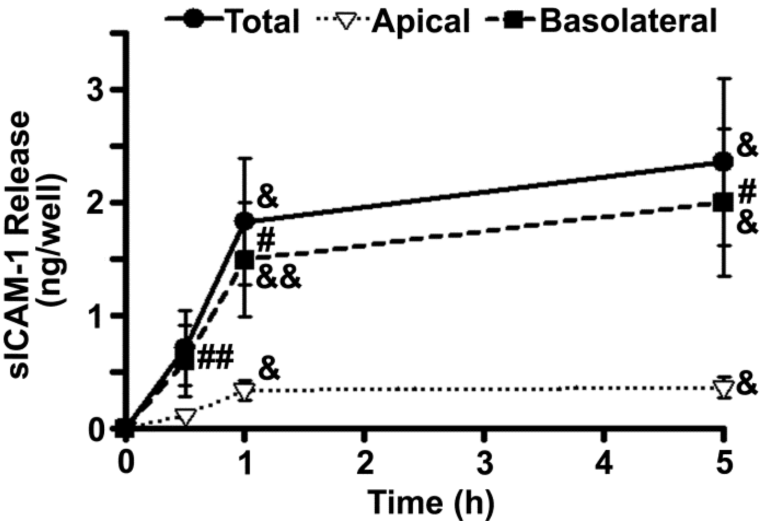

Figure S3. Cumulative distribution of sICAM-1 release by TNFα-activated HUVECs grown on transwell inserts and incubated with anti-ICAM NCs for 30 min (pulse), followed by removal of non-bound NCs and incubation in carrier-free medium for additional time up to 1 h or 5 h (chases). Data are mean ± SEM. #Comparison between apical and basolateral chambers at each time point; &comparison to 30 min (one symbol is $p < 0.1$ by Student's *t*-test and two symbols is $p < 0.1$ by Mann-Whitney Rank Sum test).

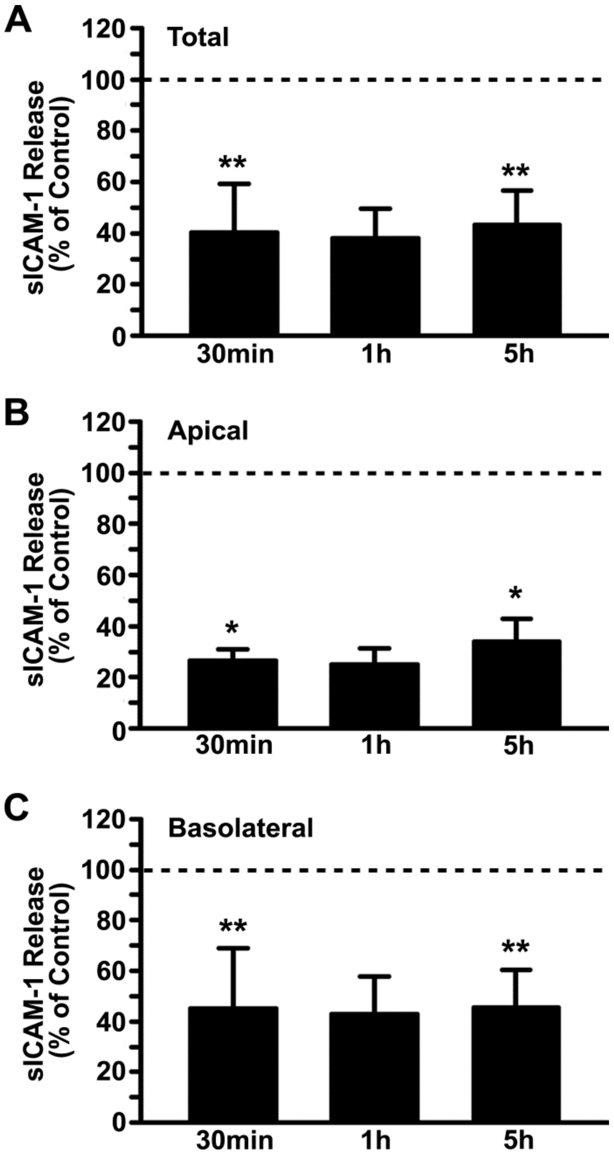

Figure S4. Cumulative distribution of sICAM-1 release by TNFα-activated HUVECs grown on transwell inserts and incubated with anti-ICAM NCs for 30 min (pulse), followed by removal of non-bound NCs and incubation in carrier-free medium for additional time up to 1 h or 5 h (chases). Data show sICAM-1 release as a percentage of cells incubated in the absence of anti-ICAM NCs (control; horizontal dashed line). Data are mean ± SEM. *Comparison to control (one symbol is $p < 0.1$ by Student's *t*-test and two symbols is $p < 0.1$ by Mann-Whitney Rank Sum test).
